# Cerebellar synapse properties and cerebellum-dependent motor and non-motor performance in *Dp71*-null mice

**DOI:** 10.1242/dmm.033258

**Published:** 2018-07-10

**Authors:** Romain Helleringer, Delphine Le Verger, Xia Li, Charlotte Izabelle, Rémi Chaussenot, Mehdi Belmaati-Cherkaoui, Raoudha Dammak, Paulette Decottignies, Hervé Daniel, Micaela Galante, Cyrille Vaillend

**Affiliations:** 1 Molecules and Circuits Department, Paris-Saclay Institute of Neuroscience (Neuro-PSI), UMR 9197, Université Paris Sud, CNRS, Université Paris Saclay, 91405 Orsay, France; 2 Cognition and Behavior Department, Paris-Saclay Institute of Neuroscience (Neuro-PSI), UMR 9197, Université Paris Sud, CNRS, Université Paris Saclay, 91405 Orsay, France

**Keywords:** Dystrophin, Dp71, Purkinje neuron, Glutamatergic transmission, Motor coordination, Cerebellum, Cognitive deficit, Brain

## Abstract

Recent emphasis has been placed on the role that cerebellar dysfunctions could have in the genesis of cognitive deficits in Duchenne muscular dystrophy (DMD). However, relevant genotype-phenotype analyses are missing to define whether cerebellar defects underlie the severe cases of intellectual deficiency that have been associated with genetic loss of the smallest product of the *dmd* gene, the Dp71 dystrophin. To determine for the first time whether Dp71 loss could affect cerebellar physiology and functions, we have used patch-clamp electrophysiological recordings in acute cerebellar slices and a cerebellum-dependent behavioral test battery addressing cerebellum-dependent motor and non-motor functions in *Dp71-*null transgenic mice. We found that Dp71 deficiency selectively enhances excitatory transmission at glutamatergic synapses formed by climbing fibers (CFs) on Purkinje neurons, but not at those formed by parallel fibers. Altered basal neurotransmission at CFs was associated with impairments in synaptic plasticity and clustering of the scaffolding postsynaptic density protein PSD-95. At the behavioral level, *Dp71*-null mice showed some improvements in motor coordination and were unimpaired for muscle force, static and dynamic equilibrium, motivation in high-motor demand and synchronization learning. *Dp71*-null mice displayed altered strategies in goal-oriented navigation tasks, however, suggesting a deficit in the cerebellum-dependent processing of the procedural components of spatial learning, which could contribute to the visuospatial deficits identified in this model. In all, the observed deficits suggest that Dp71 loss alters cerebellar synapse function and cerebellum-dependent navigation strategies without being detrimental for motor functions.

## Abstract (precis)

**Summary:** Dp71 is the most prominent dystrophin gene product in the adult brain. Here, multiple approaches including behavioral tests and electrophysiology are adopted to explore the role of Dp71 in the cerebellum.

## INTRODUCTION

The Duchenne muscular dystrophy (*dmd*) gene has at least 8 promoters driving expression of distinct dystrophin proteins that are components of different multiprotein complexes linking cytoskeletal actin to cell membrane proteins in a variety of tissues. The first three promoters consist of spliced unique first exons that control the synthesis of the 427 kDa full-length forms of the dystrophin protein (Dp427) expressed in brain, muscle or cerebellar Purkinje neurons, respectively. Five downstream promoters give rise to shorter dystrophin products (Dp260, Dp140, Dp116 and Dp71) with cell-specific expression in various organs including the nervous system (Perronnet and Vaillend, 2010; Hendriksen et al., 2015). Mutations in the *dmd* gene are responsible for Duchenne muscular dystrophy (DMD) syndrome, in which muscular degeneration is also associated with cognitive deficits presumably owing to the loss of dystrophin gene products that are normally expressed in the brain (Kim et al., 1995; Desguerre et al., 2009). The severity of the cognitive impairment in DMD is highly variable depending on the mutation location. Mutations in the first exons that selectively affect expression of Dp427 lead to moderate cognitive deficits, whereas in about one-third of patients downstream mutations inducing a cumulative loss of the shorter dystrophin products are responsible for moderate to severe intellectual disability (Desguerre et al., 2009). Dp71 loss appears to be a pivotal aggravating factor, as patients with impaired expression of Dp71 are most severely affected and systematically show intelligence quotients (IQs) below 70 (Daoud et al., 2009a).

Dp71 is the most prominent dystrophin gene product in the adult brain. The role of Dp71, as well as the functional outcome of Dp71 deficiency, appears to be quite complex owing to its expression in both neuronal and glial cells (Tadayoni et al., 2012; for a review). Dp71 was detected in postsynaptic densities and its deficiency associated with altered hippocampal glutamatergic synapse structure, synaptic transmission and plasticity (Blake et al., 1999; Daoud et al., 2009b). Moreover, electron microscopy analyses revealed that *Dp71*-null mice display significant changes in the density and size of presynaptic vesicles in hippocampal glutamatergic synapses, suggesting synapse ultrastructural alterations (Miranda et al., 2011). Dp71 is also highly expressed in glial cells where it contributes to the membrane clustering of potassium (Kir4.1) and aquaporin (AQP4) channels in perivascular domains; thus, loss of Dp71 might affect extracellular K^+^ buffering and water balance, which might also have an effect on neuronal functions (Dalloz et al., 2003; Amiry-Moghaddam et al., 2004; Connors et al., 2004; Haenggi et al., 2006; Fort et al., 2008). Dp71 probably has such a role in various glial cell types, including cerebellar Bergmann glial cells where it is required for proper localization of a pool of AQP4 water channels (Nicchia et al., 2008). However, a functional evaluation of the role of Dp71 in this structure is still lacking.

In the present study, we focus on the functional effect of Dp71 loss in cerebellar tissues, as a crucial role for cerebellum dysfunction has been highlighted in clinical studies of pediatric DMD patients (Cyrulnik and Hinton, 2008). It is now well acknowledged that cerebellar neuronal networks, particularly the climbing and parallel-fiber inputs to Purkinje neurons, contribute to navigation in rodents (Rondi-Reig et al., 2002; Rochefort et al., 2011) and their dysfunction could therefore contribute to the spatial-learning deficits reported in mice lacking Dp71 (Daoud et al., 2009b). Moreover, cerebellar alterations are commonly associated with motor deficits that could contribute to the cardinal muscular symptoms in DMD, as suggested by other mouse models with altered dystrophin-related mechanisms (e.g. Grady et al., 2006). To test these hypotheses, we first used whole-cell patch-clamp recordings in acute cerebellar slices of *Dp71*-null and wild-type (WT) littermate mice to decipher the electrophysiological properties of the two main glutamatergic inputs to cerebellar Purkinje neurons, the parallel fibers (PFs) and the climbing fibers (CFs). This approach was complemented by a quantitative immunofluorescence analysis of postsynaptic density protein 95 (PSD-95) clusters, to determine putative changes in synapse structural and molecular organization. A behavioral study of these mice was then undertaken to characterize their performance in cerebellum-dependent motor functions, including basal static versus dynamic coordination, performance during fine-timing motor learning in the rotarod, and performance in a goal-oriented task in the water maze to assess procedural learning performance and navigation strategies (Steinmayr et al., 1998; Leggio et al., 1999; Rondi-Reig et al., 2002).

## RESULTS

### Electrophysiological properties of the PF-Purkinje cell synapse

Purkinje neurons receive glutamatergic inputs from both PFs and CFs. PFs are granule cell axons that run parallel to the Purkinje cell (PC) layer in the coronal plane and form several ‘*en passant’* synapses on PC dendrites. CF inputs originating from inferior olive are strikingly different: a PC receives several synaptic contacts from a single CF, and transmission in this pathway is one to one. To assess the physiological consequences of Dp71 loss in cerebellar cortex, we therefore investigated potential alterations in excitatory postsynaptic currents (EPSC) evoked by electrical stimulation of PFs or CFs.

**Fig. 1.**
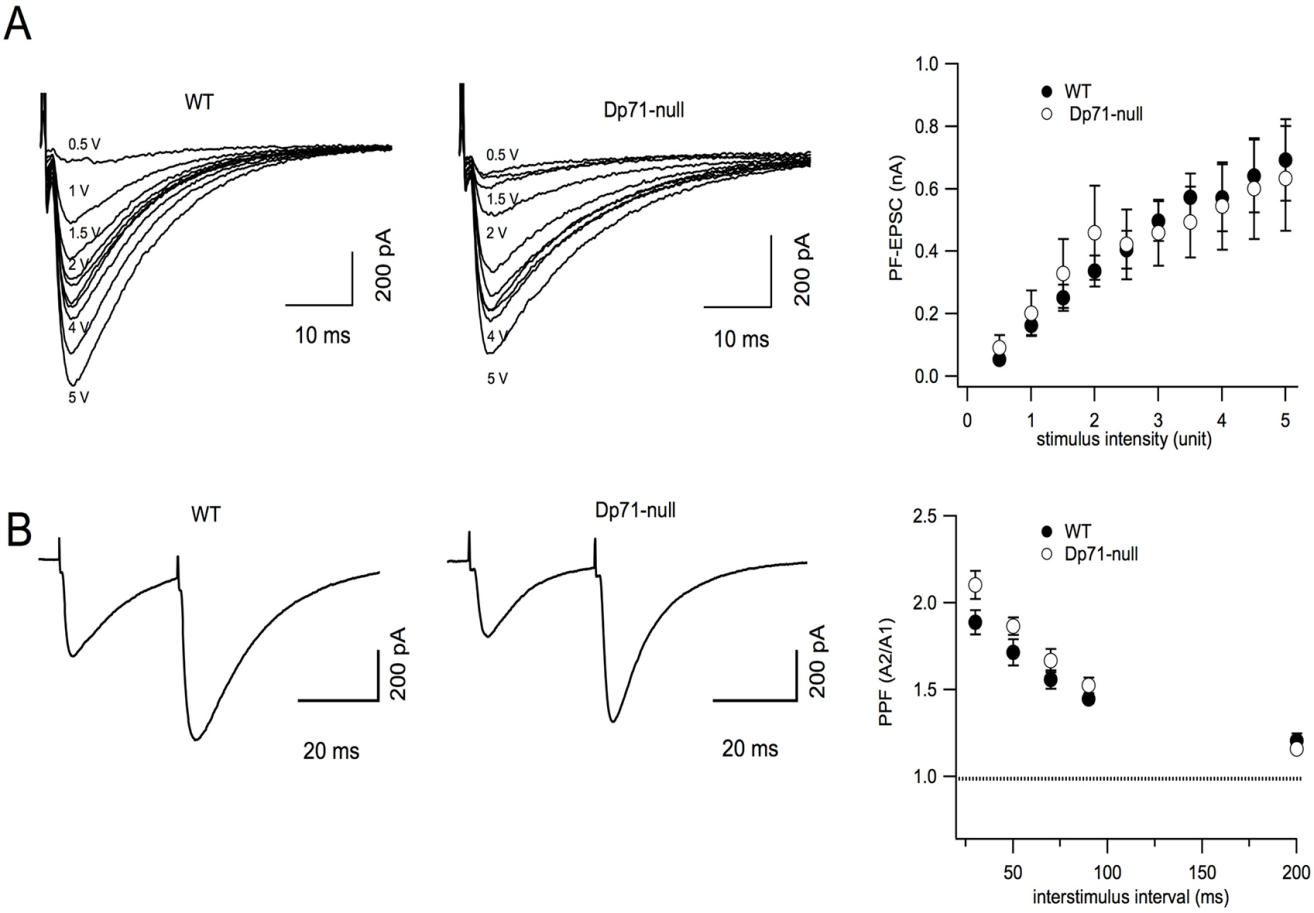
**Glutamate transmission at** **PF-Purkinje neuron synapses in WT and** ***Dp71*-null mice**. (A) Left: Representative superimposed EPSCs recorded in Purkinje neurons at increasing electrical stimulation of PFs (PF-EPSCs) in WT (filled circles) and *Dp71*-null mice (open circles). Right: Input-output relations for WT (*n*=7 cells, 5 mice) and *Dp71*-null mice (*n*=9 cells, 5 mice); *P*>0.45. (B) Left: PF-EPSCs evoked by two stimuli separated by 30 ms induced PPF in both WT (filled circles) and *Dp71*-null mice (open circles). Right: mean values of PPF calculated at different ISI (30, 50, 70, 90 and 200 ms) for WT (*n*=9 cells, 6 mice) and *Dp71*-null mice (*n*=8 cells, 5 mice; *P*>0.08). Holding potential was −70 mV with gabazine 5 µM in the extracellular solution.

EPSCs were identified as PF-induced EPSCs (PF-EPSCs) when their amplitude increased in a graded manner with stimulus intensity and when they displayed paired-pulse facilitation (PPF) (Fig. 1A,B). PF-EPSCs in WT (*n*=7 cells, 5 mice) and in *Dp71*-null (*n*=9 cells, 5 mice) mice presented similar amplitudes as a function of stimulus intensity, as shown by the superimposed input-output curves of the two genotypes (all *P*>0.14; Fig. 1A). PF-EPSCs in WT and *Dp71*-null mice presented similar kinetics, as revealed by the mean rise time (1.96±0.30 ms in WT and 1.67±0.14 ms in *Dp71*-null mice; *P*>0.5, *n*=13 cells, 10 mice) and decay time constant (32.13±2.69 ms vs 27.83±3.94 ms; *P*>0.09, *n*=13 cells, 9 mice). PF-EPSCs were completely blocked by addition of the α-amino-3-hydroxy-5-methyl-4-isoxazole propionic acid (AMPA) receptor antagonist 2,3-dioxo-6-nitro-1,2,3,4-tetrahydrobenzo[*f*]quinoxaline-7-sulfonamide (NBQX) (10 µM), confirming that these currents were entirely mediated by the activation of AMPA/Kainate receptors (data not shown). To investigate release probability at PF-PC synapses, two consecutive stimuli were delivered with different interstimulus intervals (ISI: 30, 50, 70, 90 and 200 ms) and the ratio between the amplitude of the second and the first EPSC was calculated (paired-pulse ratio). The PPF observed at each ISI was not significantly different between WT and *Dp71*-null mice (all *P*>0.07; *n*=9 cells, 6 WT mice versus *n*=8 cells, 5 *Dp71*-null mice; Fig. 1B).

### Electrophysiological properties of the CF-Purkinje cell synapse

**Fig. 2.**
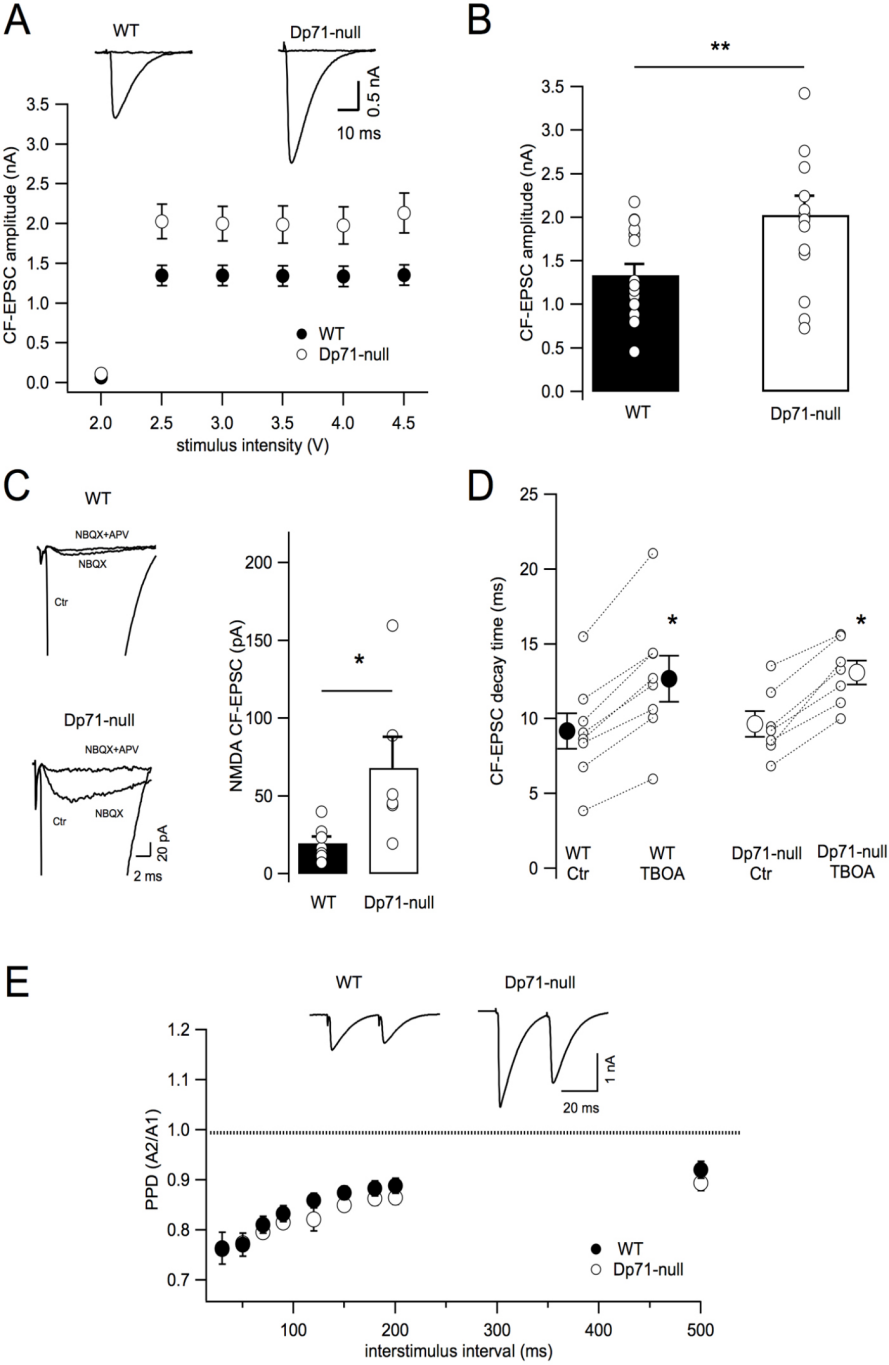
**Purkinje neurons from *Dp71*-null mice present larger CF-EPSCs.** (A) Representative ‘all or none’ CF-EPSCs from a Purkinje neuron in WT (filled circles) and *Dp71*-null mice (open circles). The graph shows CF-EPSC mean amplitudes as a function of stimulus intensity for *n*=16 cells in WT (11 mice) and *n*=15 cells in *Dp71*-null mice (11 mice). (B) Distribution and mean peak values of CF-EPSCs from WT (*n*=16 cells) and *Dp71*-null mice (*n*=15 cells; *P*=0.01). (C) Left: CF-EPSCs are mainly mediated by activation of AMPA/Kaïnate receptors, as they are strongly inhibited by NBQX (10 µM). The remaining current is partially abolished by further addition of APV (50 µM) indicating a role of NMDA receptors. Right: Individual values of the NMDA receptor component of CF-EPSC in WT (*n*=7 cells, 5 mice) and *Dp71*-null mice (*n*=6 cells, 4 mice; *P*=0.029). (D**)** TBOA (100 µM) significantly increases the decay time of CF-EPSC in both WT (*n*=8 cells, 4 mice; *P*=0.01) and in *Dp71*-null (*n*=7 cells, 3 mice; *P*=0.02) mice and no statistical difference was observed in the two populations (*P*=0.71). (E) Top: CF-EPSCs evoked by two stimuli separated by 30 ms show a PPD in both WT and *Dp71*-null mice. Bottom: Time course of recovery from PPD is similar between WT and *Dp71*-null mice. Mean PPD values are from *n*=9 Purkinje cells in both WT and *Dp71*-null mice. ISIs were fixed at 30, 50, 70, 90, 120, 150, 180, 200 and 500 ms (*P*>0.43 for each interval). Holding potential was −20 mV with gabazine 5 µM in the extracellular solution.

To study CF-induced EPSCs (CF-EPSCs), PC membrane potential was held at −20 mV (see Materials and Methods section) in order to reduce the driving force for glutamate currents and to limit the voltage clamp escape generated by the large CF-EPSCs. In this condition, CF-EPSCs were distinguished from PF-EPSCs on the basis of their ‘all or none’ response (Fig. 2A) and by the occurrence of paired-pulse depression (PPD) when two stimuli were delivered (Fig. 2E). CF-EPSCs in WT animals were characterized by a mean peak amplitude of 1.35±0.13 nA (*n*=16 cells, 10 mice). Increasing stimulation intensity left the CF-EPSC amplitude unchanged, thus indicating that PCs were contacted by only one CF (Fig. 2A). We only observed a double CF innervation in 1 out of 6 Purkinje neurons, confirming the low percentage of multiple innervated PCs in adult mice (Nishiyama and Linden, 2004). In *Dp71*-null mice, CF-EPSC amplitudes were significantly larger than in WT PCs, with a mean peak amplitude of 2.0±0.2 nA (*n*=16 cells, 12 mice, *P*<0.02; Fig. 2A,B). This enhancement in *Dp71*-null CF-EPSC peak amplitudes was not explained by an increase in the number of CFs innervating a given PC, as cells from *Dp71*-null mice also presented the typical ‘all or none’ response as in WT (Fig. 2A) and only 2 PCs out of 7 presented a double CF innervation. To investigate the nature of this enhanced CF-EPSC, we pharmacologically blocked AMPA/Kainate receptors that mediate the majority of CF responses in Purkinje neurons (Dzubay and Otis, 2002). As shown in Fig. 2C (top traces), NBQX (10 µM) drastically reduced CF-EPSC amplitude, leaving a residual current of only 19.7±4.2 pA (*n*=7 cells, 5 mice). This small NBQX-resistant current had the same all or none properties as the full-size CF-EPSC and was partly suppressed by the addition of 50 µM DL-2-amino-5-phosphonovaleric acid (APV), a selective *N*-methyl-D-aspartate (NMDA) receptor antagonist (57.03±16.08% inhibition, *n*=3 cells, 3 mice; Fig. 2C, top traces). In *Dp71*-null mice, the NMDA component of CF-EPSC was significantly enhanced when compared with WT mice (68±20 pA, *n*=6 cells, 4 mice, *P*<0.02; Fig. 2C) and APV blocked that current by 55.9±18.2%, as in WT animals (*n*=5 cells, 3 mice, *P*>0.05). These data show that in *Dp71*-null mice the enhanced CF-EPSC amplitude could be observed for both AMPA/Kainate and NMDA receptor components. However, the inhibitory effect of NBQX on CF-EPSC amplitude was similar in *Dp71*-null and WT mice (95.1±1.4%, *n*=6 cells and 97.9±0.6%, *n*=7 cells, respectively; *P*>0.09), suggesting that there was no imbalance in the AMPA/NMDA ratio in *Dp71*-null mice. Finally, as glutamate uptake has an important role in modulating extracellular glutamate concentration, we analyzed the contribution of glutamate transporters to CF-EPSCs (Fig. 2D). The mean decay time constant of CF-EPSC was 9.17±1.9 ms in WT (*n*=8 cells, 4 mice) and 9.66±0.85 ms in *Dp71*-null mice (*n*=7 cells, 3 mice) (both *P*>0.7). Inhibition of glutamate transporters by TBOA (100 µM) significantly increased this parameter in both WT (to 140.3±3.7%, *n*=8 cells, *P*<0.02; Fig. 2D) and *Dp71*-null mice (137.7±6.1%, *n*=7 cells, *P*<0.02; Fig. 2D) in a similar manner (both *P*>0.5). TBOA also slightly increased both the amplitude and rise time of CF-EPSCs (data not shown), but again no statistical difference was observed between the two genotypes for these parameters (peak amplitude *P*>0.2; rise time *P*>0.5). Finally, we measured the PPD, a presynaptic form of short-term plasticity that characterizes CF-EPSC. As shown in Fig. 2E, there was no significant difference between WT (*n*=9 cells, 5 mice) and *Dp71*-null mice (*n*=9 cells, 6 mice) at any interstimulus interval (all *P*>0.4).

### Plasticity of CF-PC synapses

**Fig. 3.**
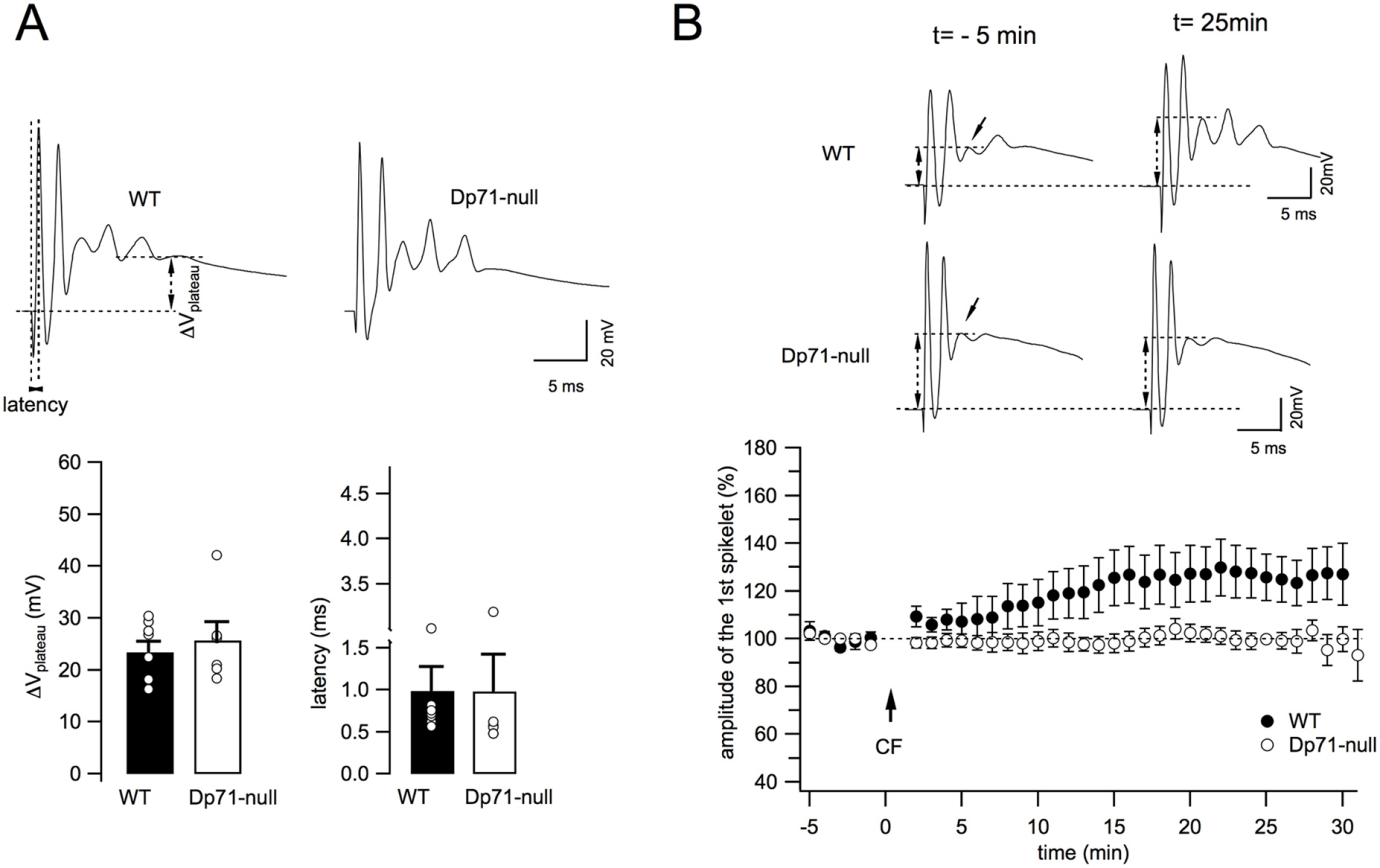
**Tetanic stimulation of CF-PC synapses reveals long-term plasticity alterations in *Dp71*-null mice.** (A) Top: Representative complex spikes recorded in a WT (left) and *Dp71*-null (right) PC following CF stimulation at 0.033 Hz. Note the initial fast depolarizing component followed by smaller and slower depolarizing peaks (spikelets). Dashed lines indicate the parameters characterizing the complex spike: the depolarized plateau potential after the last spikelet (ΔV_plateau_) and the time interval between the stimulus artifact and the peak of the first spikelet (latency). Single and averaged values from WT (*n*=8 cells, 3 mice) and *Dp71*-null (*n*=6 cells, 2 mice) mice are reported below. *P*>0.5 for all. (B) Top: CF-evoked complex spikes before (t=−5 min) and after CF stimulation at 5 Hz for 30 s (t=25 min) in WT and mutant mice. Bottom: Time course of the amplitude of the first spikelet in WT (*n*=7 cells, 3 mice) and *Dp71*-null mice (*n*=6 cells, 2 mice). The arrow shows the moment of CF tetanic stimulation. Each data point represents the average of two successive responses evoked at 0.033 Hz.

In PCs, CF activation triggers the discharge of a multicomponent spike characterized by a fast, classic Na^+^ action potential followed by smaller but broader spikes called spikelets, which are due to the activation of voltage-dependent Ca^2+^ conductances (Fig. 3A). CF-triggered complex spikes generate prominent dendritic Ca^2+^ increases in PCs mediating several types of long-term plasticity at PF synapses (Ohtsuki et al., 2009; Daniel and Crepel, 2012; Hoxha et al., 2016). Moreover, homosynaptic long-term depression (LTD) occurs at CF-PC synapses (Hansel and Linden, 2000), thus confirming the widespread notion that CF activity governs various types of plasticity that can modulate PC output (Ohtsuki et al., 2009). As these forms of synaptic plasticity involve glutamatergic transmission, we hypothesized that the enhanced neurotransmission at CF-PC synapses in *Dp71*-null mice could have an effect on long-term plasticity at these same synapses. An initial set of experiments was conducted to determine whether the complex spike waveform elicited by CF stimulation presented similar properties in WT and *Dp71*-null mice. Although there was a certain degree of variability from cell to cell, the number of spikelets (3.5±0.6, *n*=6 cells in WT vs 3.0±0.4 in *Dp71*-null mice, *P*>0.5; data not shown), the plateau potential and the latency were globally similar in WT (*n*=8 cells, 3 mice) and mutant mice (*n*=6, 2 mice) (all *P*>0.5; Fig. 3A), suggesting that the ∼50% increase in CF-EPSC amplitude found in *Dp71*-null mice (see Fig. 2A,B) does not affect the production of multicomponent spikes. We then induced synaptic plasticity at CF-PC synapses by stimulating CFs at 5 Hz for 30 s. Although this stimulation protocol was previously shown to reduce the amplitude of CF-EPSC (in voltage clamp) and/or that of the first spikelet (in current clamp) in young rats (Hansel and Linden, 2000; Weber et al., 2003; Schmolesky et al., 2007), in our hands using adult WT mice, this protocol elicited a progressive increase in the amplitude of the first spikelet that stabilized ∼15 min after the stimulation train (Fig. 3B). During the last 5 min of our recordings, from 25 to 30 min following CF tetanization, the amplitude of the first slow spikelet increased to 126.1±9.6% of baseline (*n*=7 cells, *P*<0.05; Fig. 3B). In marked contrast, CF tetanization did not induce any noticeable change in spike amplitude in *Dp71*-null mice, as amplitude of the first spikelet was 100.6±3.6% of baseline at 25-30 min post-tetanus (*n*=6 cells, *P*>0.5; Fig. 3B). This observation indicates that, in addition to the large enhancement of basal neurotransmission, the absence of Dp71 in these mice also impaired synaptic plasticity at the CF synapse of cerebellar PCs.

### Excitatory synapse organization

**Fig. 4.**
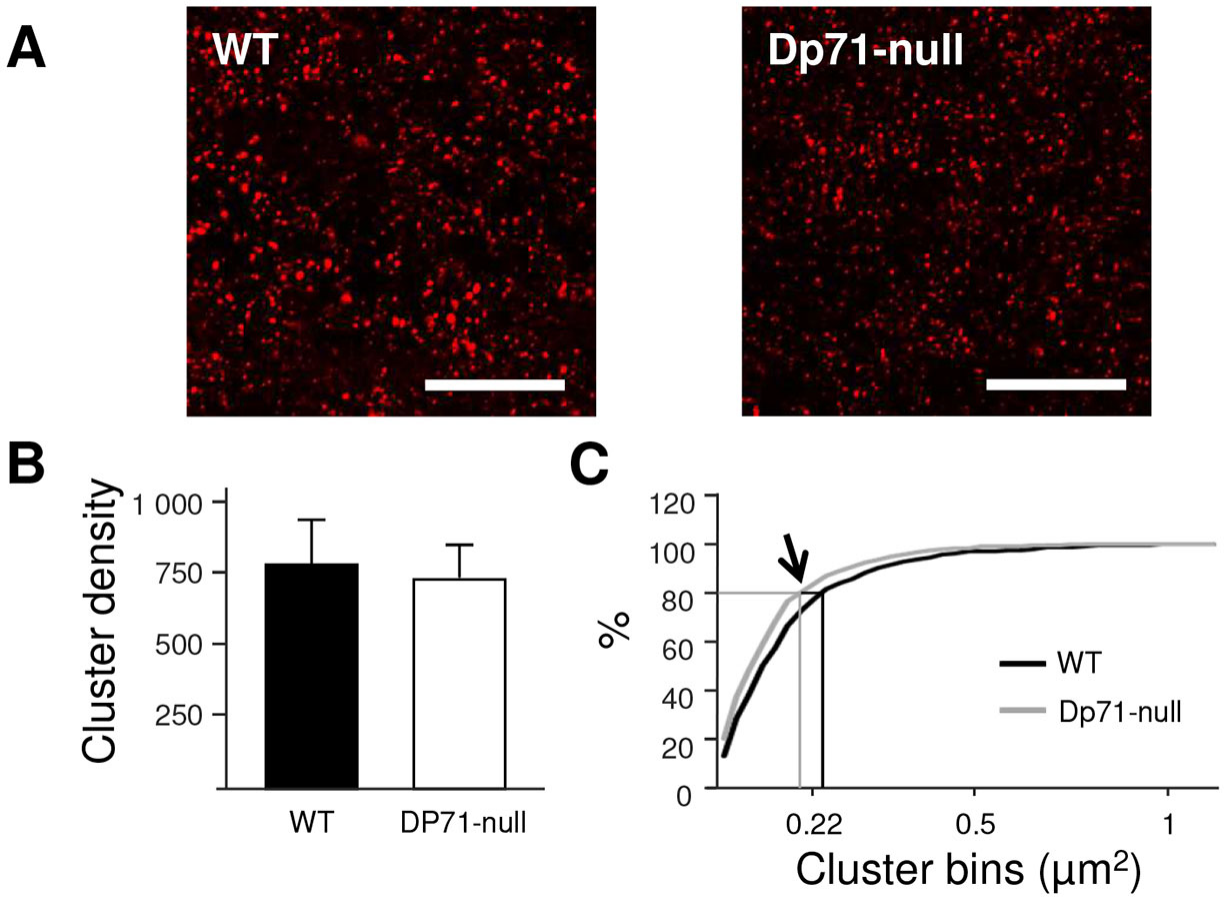
**Excitatory synapse organization.** (A) Sample confocal images taken in the cerebellar molecular layer in WT and *Dp71*-null mice. Immunoreactive clusters of PSD-95 can be seen in the proximal dendritic area of PCs (≤60 µm from neuron soma), as the majority of CF synapses occupy this territory on the PC dendritic tree. Scale bar: 20 µm. (B) Density of PSD-95 clusters in the Purkinje neuron proximal dendritic area, normalized to 10,000 µm^2^ of tissue. (C) Cluster sizes were analyzed in a cumulative frequency curve. The distribution of PSD-95 clusters (0.05-2 µm^2^) presented a leftward shift in *Dp71*-null mice (arrow, *P*<0.005, *n*=3 mice per genotype). The lines show that 80% of clusters in *Dp71*-null mice were ≤0.220 µm^2^, while 80% were ≤0.269 µm^2^ in WT mice.

Synaptic expression of the scaffolding postsynaptic density protein PSD-95 was characterized as punctate immunofluorescent dots (clusters) scattered in the dendritic areas of PCs in the molecular layer (Fig. 4A). As our electrophysiological results indicate that the neurotransmission at CF-PC synapses is altered in *Dp71*-null mice (Figs 2 and 3), the density and size of PSD-95 clusters was analyzed in a proximal dendritic area (<60 µm from PC soma; Fig. 4A) to specifically target the territory occupied by CF synapses on the PC dendritic tree (Strata and Rossi, 1998; Xu-Friedman et al., 2001; Ichikawa et al., 2016). As shown in Fig. 4B, the density of PSD-95-positive synapses was comparable between the two genotypes (*P*>0.8). However, there was a significant leftward shift of the distribution curves of PSD-95 cluster sizes in *Dp71*-null mice compared with WT mice (Kolmogorov-Smirnov test, *P*<0.005; Fig. 4C), indicating a higher proportion of small clusters in *Dp71*-null mice.

### Motor abilities and exploratory behavior

**Fig. 5.**
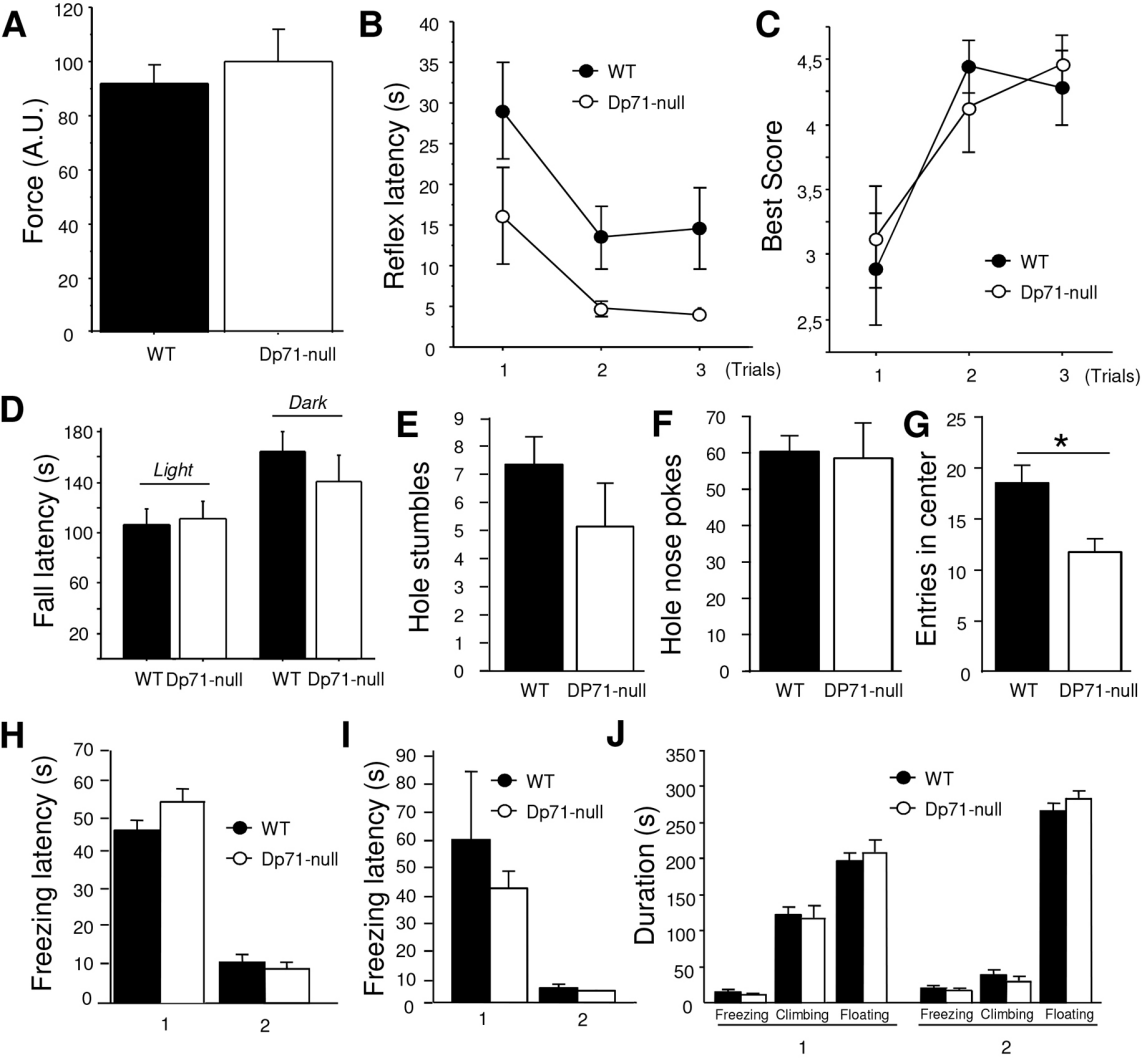
**Muscle force, coordination and motivation.** (A) Force expressed in arbitrary units (A.U.) averaged from three successive trials in the grip strength test (9 WT and 7 *Dp71*-null mice). (B,C) Performance during the three trials of the wire suspension test (18 WT, 15 *Dp71*-null mice) expressed as the reflex latency to touch the wire with one hind paw (B) and the best score (C) according to the motor performance scale (Aruga et al., 2004). (D) Latency to fall from the unstable platform in light (mean of three successive trials) and dark conditions (one trial, 24 h later) (*n*=10 per genotype). (E-G) Exploration in the hole board showing the number of stumbles (E), number of nose pokes in holes (F) and number of entries in the virtual central area of the apparatus (G). *P<0.01, one-way ANOVA. (H) Freezing latency in two successive training days (1, 2) in the tail suspension test (13 WT, 14 *Dp71*-null mice). (I) Freezing latency in two successive training days (1, 2) in the forced swim test. (J) Duration of freezing, climbing and floating in two successive training days (1, 2) in the forced swim test (*n*=14 per genotype).

All mice of both genotypes reached maximal criterion by maintaining a grip for 2 min in three successive trials in the inverted screen test (data not shown). Muscular force was also comparable between genotypes in the grip strength test (*P*>0.5; Fig. 5A). In the wire suspension test, the latency to touch the wire with one hind paw was shorter in *Dp71*-null compared with WT mice (*P*<0.05; Fig. 5B), suggesting facilitation of this reflex that involves coordination of forepaw traction and hind paw flexion. There was no genotype difference in the latency to fall (*P*>0.1), however, or to escape to one of the wire supports in any trial (*P*>0.4), with no significant genotype×trial effect. Finally, mice of both genotypes reached comparable best scores (Fig. 5C) according to the motor performance scale (Aruga et al., 2004). Static balance was evaluated by placing mice on an unstable platform, to evaluate their capacity to maintain balance when displacements are limited. This was first tested in light and then in complete darkness to force the mice to use vestibular-associated self-motion cues in the absence of visual cues. *Dp71*-null and WT mice showed comparable fall latencies in both conditions, suggesting unaltered vestibular functions (Fig. 5D). The frequency of slips recorded in light conditions were also comparable between genotypes (*Dp71*-null 0.059±0.19; WT 0.044±0.011; *P*>0.4). During exploration of a hole board, mice of both genotypes made a comparable number of stumbles in holes during walking (*P*>0.2; Fig. 5E), travelled similar total distances (*P*>0.2), and exhibited comparable average and maximal travelling speeds (*P*>0.1), suggesting unaltered dynamic balance and motor coordination in *Dp71*-null mice while in motion. No specific stereotypies could be detected in the transgenic mice in this test (number of hole nose pokes, genotype effect *P*>0.8; Fig. 5F). In the central area of the apparatus, however, the *Dp71*-null mice displayed a reduced number of entries (*P*<0.01, Fig. 5G) and a shorter distance travelled (WT 2.51±0.14 m; *Dp71*-null 1.56±0.19 m; *P*<0.001), suggesting enhanced anxiety.

### Motivation in high motor-demand aversive situations

Motivation was assessed using the tail suspension (TST) and forced swim (FST) tests, two paradigms in which mice initially engage vigorous movements to escape from an inescapable stressful situation, and then show progressive increase in the frequency and duration of immobility episodes that characterize behavioral despair. In the TST, latency of the first immobility episode (freezing latency) was shorter during the second day of training (Fig. 5H) and immobility duration was conversely longer in all mice (data not shown), with no significant genotype differences (*P*>0.1, both parameters). Likewise, there was no genotype differences in the FST (*P*>0.3, all parameters). Freezing latency (Fig. 5I) and amount of climbing behavior (Fig. 5J) drastically decreased between the 2 days of testing in both genotypes, whereas freezing and floating durations were increased during the second day (Fig. 5J).

### Motor synchronization learning

**Fig. 6.**
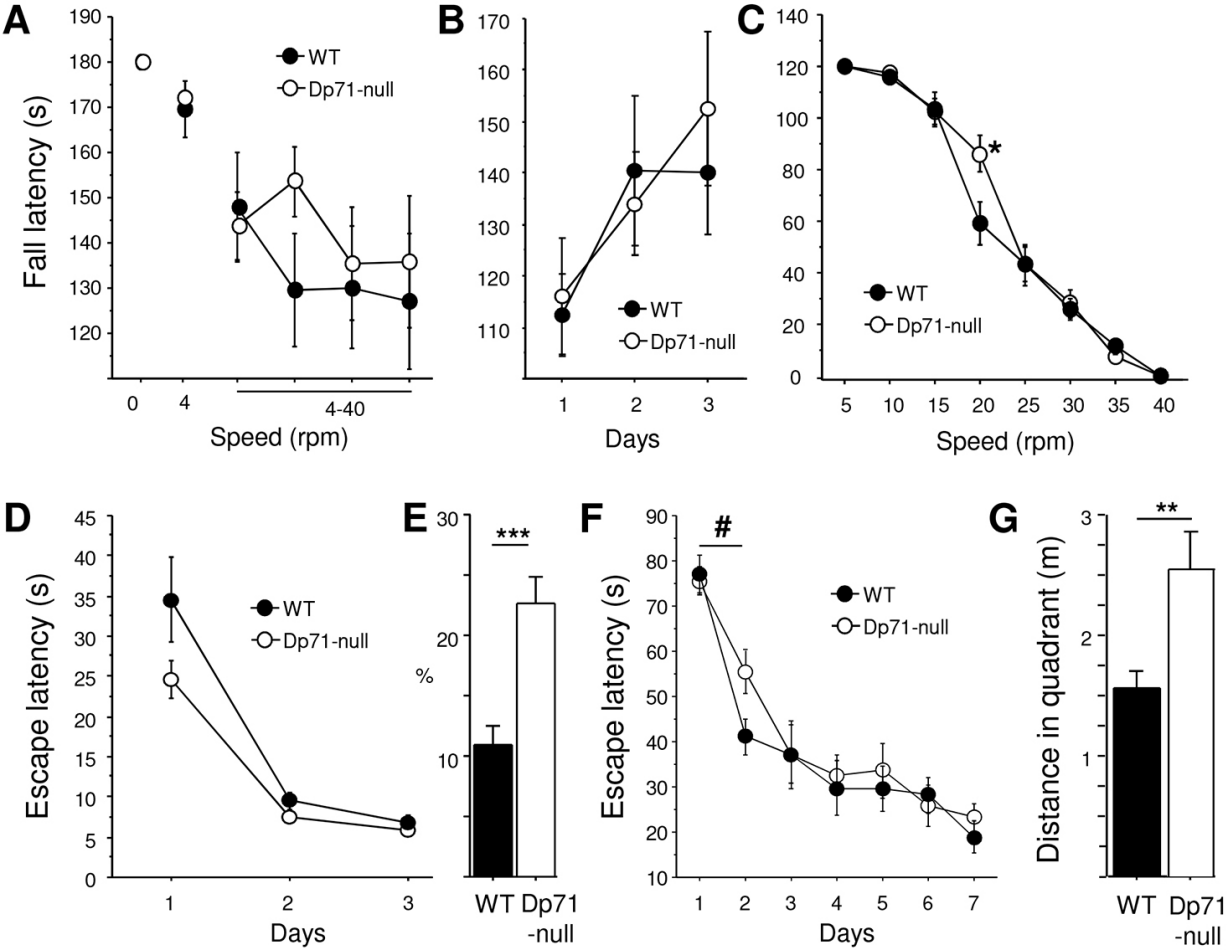
**Motor synchronization learning and navigation strategies.** (A,B) Motor synchronization learning in the rotarod test. In all experiments, performance was quantified as the latency to fall from the rod. In a first protocol (A), mouse equilibrium was evaluated following placement of the mouse on the non-rotating rod (speed=0), and then on the rod rotating at a constant low speed (4 rpm) and during accelerating speed ramps (4-40 rpm) during 4 days (18 WT and 15 *Dp71*-null mice). In a second protocol (B,C), motor learning was first assessed during accelerating speed ramps during 3 days (B) and then in eight successive trials, each at a single constant speed as indicated on the *x*-axis (C) (*n*=10 per genotype). (D) Escape latency during 3-day training in the visible-platform protocol in a water maze (10 WT, 11 *Dp71*-null mice). (E) Percentage time spent in the Whishaw's corridor during the first training day in the visible-platform protocol. (F) Escape latency during 7-day training in the non-visible-platform protocol with fixed departure locations in a water maze (*n*=12 per genotype). (G) Distance swum in the target quadrant before finding the platform during day 2. Significant genotype effects: *P<0.05, **P<0.01, ***P<0.001. ^#^*P*<0.05, significant genotype×day interaction.

Motor synchronization learning was further detailed using the rotarod apparatus. In a first set of experiments, mice were successively placed on the non-rotating rod, on the rod rotating at a constant low speed (4 rpm) and then during 4 consecutive days on a rotating rod that accelerated from 4 to 40 rpm (Fig. 6A). Mice showed normal static equilibrium as they did not fall from the non-rotating rod. The latency to fall from the rotating rod was also comparable between genotypes, whether at low constant speed or during an accelerating speed ramp (*P*>0.5). *Dp71*-null mice thus demonstrated normal ability to anticipate and prevent fall by synchronizing walk with the speed of the rod to maintain balance. In a second experiment, mice were first submitted to accelerating speed ramps (Fig. 6B), during which mice displayed increasing performance across 3 training days reflecting rapid learning of the coordination motor task (*P*<0.01). No differences were found between genotypes during learning (genotype and genotype×day interaction, *P*>0.5). On the fourth day, mice were tested 4 times in 8 consecutive trials at constant speeds of 40, 35, 30, 25, 20, 15, 10 and 5 rpm, respectively (Fig. 6C). Performance of *Dp71*-null mice was globally comparable to that of the WT mice (genotype effect *P*>0.3), yet there was a significant genotype×speed interaction (*P*<0.05) owing to the better performance showed by *Dp71*-null mice at a 20 rpm speed (*P*<0.05).

### Navigation performance and strategy

Goal-oriented navigation strategies were analyzed in two different paradigms and the main genotype differences are summarized in Table 1. Visual guidance strategy was first assessed in a water maze with a visible platform (proximal cue). In this test, mice were successively released from different start points at the periphery of the maze. The time needed to find the platform (escape latency; Fig. 6D) and the distance swum to platform (data not shown) rapidly decreased across trials in both genotypes. There was a trend in *Dp71*-null mice to display shorter escape latencies compared with WT mice (*P*=0.054; Fig. 6D), yet this was mostly apparent on the first day of training (*P*=0.08). Interestingly, however, *Dp71*-null mice also had a trend to spend less time circling along walls (WT 7.78±1.39 s; *Dp71*-null 5.12±0.9 s; *P*=0.057), whereas they displayed significantly shorter cumulative distance to platform (WT 9.97±2.04 ms; *Dp71*-null 6.71±1.10 ms; *P*<0.05) and shorter corrected integrated path length (WT 8.29±2 ms; *Dp71*-null 5.19±1.07 ms; *P*<0.05), two indexes of a better path efficiency. This suggests that *Dp71*-null mice made more goal-oriented paths associated with reduced exploration of the other parts of the maze that were distant from the visible platform. This was confirmed by analysis of the time spent and distance swum within a 15 cm wide Whishaw's corridor, a virtual rectangular zone that runs from the animal's start position to the platform. Indeed, the percentage time spent and percentage distance swum in this zone was significantly larger in *Dp71*-null mice during the first training day (*P*<0.001 and *P*<0.01, respectively) (Fig. 6E).

**Table 1.**
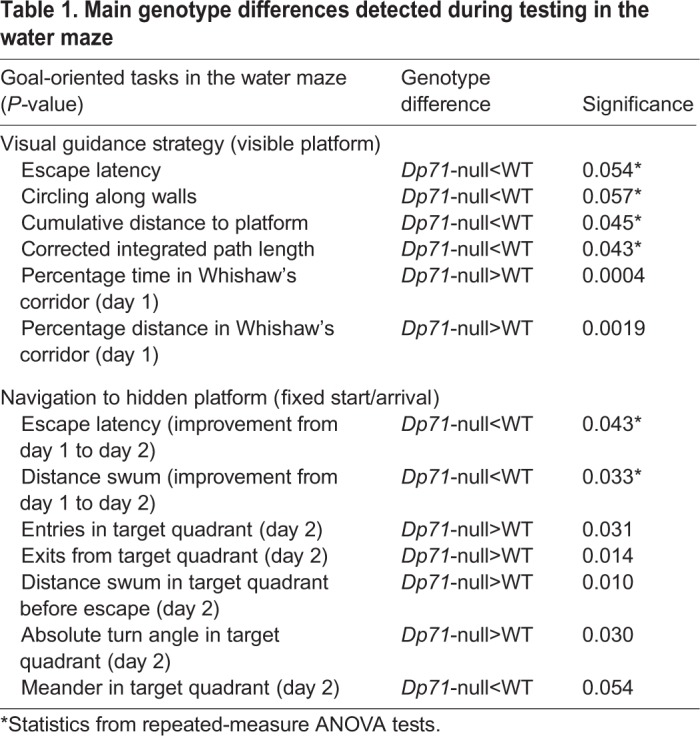
**Main genotype differences detected during testing in the water maze**

When mice had to find a fixed hidden platform from fixed start points (Fig. 6F,G; Table 1), their learning performance also rapidly reached asymptotic performance, expressed as a drastic reduction in escape latency (data not shown) and in the distance swum to reach the platform (Fig. 6F). In contrast with the visible-platform task, however, *Dp71*-null mice showed a learning delay between training days 1 and 2 (time to platform×genotype interaction *P*<0.05; distance swum×genotype *P*<0.04; indicated by # in Fig. 6F), an initial period during which mice normally quickly acquire the procedural component of the task. To detail the navigation strategy associated with this transient learning delay in *Dp71*-null mice, we analyzed several parameters reflecting path tortuosity and path efficiency during the second training day. During this session, there was no difference between genotypes regarding the time spent and distance swum in the Whishaw's corridor. However, *Dp71*-null mice made more entries in the target quadrant (WT 2.94±0.26; *Dp71*-null 3.92±0.31; *P*<0.04) and more exits from it (WT 2±0.27; *Dp71*-null 3.27±0.35; *P*<0.02), as compared with WT mice; thus, *Dp71*-null mice swam longer distances in the target quadrant before finding the platform (*P*<0.01; Fig. 6G). Interestingly, when analyzed in the target quadrant, the path of *Dp71*-null mice was characterized by larger absolute turn angle (between each movement vector of the mouse) (WT 829±76; *Dp71*-null 1142±102; *P*<0.04) and slightly smaller meander (WT 607±37; *Dp71*-null 502±22; *P*=0.054) as compared with WT mice. This result suggests that path efficiency and tortuosity were altered in *Dp71*-null mice.

## DISCUSSION

Progressive muscle weakness is the most prominent, but not the only, manifestation in DMD syndrome. Indeed, this dramatic pathology is also associated with important deficits in high-order cognitive and executive functions, some of which are thought to involve the cerebellar complex, such as sentence repetition, phonological processing, verbal working memory and altered social behavior (Cyrulnik and Hinton, 2008). This hypothesis is consistent with the extensive expression in the cerebellum of both the full-length dystrophin, Dp427, and the short C-terminal product of this multipromoter gene, Dp71 (Knuesel et al., 1999; Blake and Kröger, 2000; Hendriksen et al., 2016). From a mechanistic point of view, a possible role of Dp427 in controlling γ-aminobutyric acid (GABA_A_) receptor clustering (Kueh et al., 2008; 2011), and thus in inhibitory synaptic transmission in Purkinje neurons (Anderson et al., 2003), has already been identified. By contrast, no information is available on the functions of Dp71 in cerebellar physiology. The loss of Dp71, which in DMD patients is associated with moderate to severe intellectual disability, mainly induces spatial-learning (navigation) deficits in *Dp71*-null mice (Daoud et al., 2009b). As cerebellar neuronal networks are involved in navigation strategies (Rochefort et al., 2013), we tested the hypothesis that a cerebellar dysfunction contributes to the spatial-learning deficits in these mice. As *Dp71*-null mice do not have myopathy, it also constitutes an interesting model to determine whether central neuronal alterations could contribute to the motor deficits associated with DMD.

We first examined the effect of Dp71 loss on glutamatergic synaptic transmission onto Purkinje neurons. We identified an important contribution of Dp71 to the determination of synaptic strength, selectively at the synapses formed by the climbing fibers (CF-PC), but not at the vastly more numerous parallel fibers (PF-PC). As previously reported in hippocampal CA1 glutamatergic synapses (Daoud et al., 2009b), our electrophysiological experiments revealed that both the AMPA and NMDA receptor-mediated components of CF-EPSCs were abnormally enhanced in *Dp71*-null mice compared with WT littermate control mice. These alterations could, in principle, be attributed to diverse presynaptic and/or postsynaptic factors. The lack of changes in paired-pulse synaptic depression, however, suggest that the probability of glutamate release is not modified in the absence of Dp71. Moreover, the larger CF-EPSCs recorded in *Dp71*-null mice probably do not stem from multiple CF innervation of PCs, because the percentage of PCs contacted by at least two CFs was similarly low in both WT and mutant mice. It therefore seems unlikely that major presynaptic alterations could explain enhanced neurotransmission in *Dp71*-null mice, yet a putative increase in the number of presynaptic boutons per fiber cannot be excluded. Importantly, our data show that the increased synaptic strength at CF-PC synapses concerns both the AMPA and NMDA receptor components of EPSCs. It is important to mention that, in PCs, functional NMDA receptors are expressed at CF-PC synapses, but not at PF-PC contacts. NMDA receptors in PCs are mainly formed by NR2A and NR2B subunits, which provide these cells with high sensitivity to extracellular Mg^2+^ block and have a possible role in synaptic plasticity at these synapses (Piochon et al., 2007, 2010). To further evaluate the possible consequence of this stronger CF-PC response, we therefore analyzed the induction and expression of long-term potentiation (LTP) and found that this long-lasting form of synaptic plasticity is impaired at these CF-PC synapses in *Dp71*-null mice.

The specific neurophysiological modifications identified at CF-PC synapses in *Dp71*-null mice are reminiscent of those found in the hippocampus, where enhanced neurotransmission was also associated with occlusion of LTP expression; however, the primary mechanisms linking these alterations are uncertain (Daoud et al., 2009b). It is possible that Dp71 deficiency independently alters glutamatergic synapse structure and function as well as glial-dependent extracellular ion homeostasis, which could both contribute to the modification of synaptic transmission and plasticity. The observed changes in CF-PC responses suggest that Dp71 loss altered postsynaptic mechanisms needed for proper neurotransmission, which might include a putative increase in the number of CF-PC contacts and/or glutamate receptors. To test this hypothesis, we performed a quantitative analysis of the postsynaptic density protein PSD-95 that is involved in the postsynaptic clustering of NMDA and AMPA receptors (El-Husseini et al., 2000; Xu, 2011) and which was found to interact with Dp71 in brain tissues (Daoud et al., 2009b). We analyzed the number and size of PSD-95 immunoreactive clusters, which represent glutamatergic postsynaptic compartments, in the proximal dendritic region of Purkinje cells corresponding to the main territory occupied by CF synapses (Xu-Friedman et al., 2001; Ichikawa et al., 2016). We found the density of PSD-95-positive synapses to be comparable in WT and *Dp71*-null mice. Moreover, an evaluation of AMPA receptor subunit content by western blot analysis revealed comparable levels of GluA1-3 subunits in cerebellum of the two genotypes (*P*>0.1, *n*=4 mice per genotype; R.D. and P.D., unpublished results). Overall, these data mitigate the possibility that mutant mice might display an increased number of CF contacts or higher level of expression of postsynaptic AMPA receptors. Accordingly, it is believed that Dp71 is dispensable for postsynaptic targeting of glutamate receptor complexes, but might be a modulator of their organized distribution at the postsynaptic membrane (Daoud et al., 2009b). To address this latter hypothesis we analyzed the distribution of PSD-95 cluster size and found that *Dp71*-null mice have a larger proportion of small clusters compared with WT mice. Indeed, this observation suggests that Dp71 is implicated in the molecular organization of glutamatergic postsynaptic densities. Interestingly, quantitative changes in PSD-95 expression might correlate with modifications in the length of the postsynaptic density and with the number of postsynaptic glutamate receptors, which has been associated with the ability to express synaptic plasticity (discussed in Ehrlich et al., 2007; Daoud et al., 2009b; Miranda et al., 2009). This finding suggests a possible relationship between reduced PSD-95 cluster size and impaired synaptic plasticity in *Dp71*-null mice, perhaps because lower levels of PDS-95 are not sufficient to stabilize NMDA/AMPA receptor clusters. Nevertheless, other molecular mechanisms might explain the changes in CF-PC synaptic strength and should be considered in future studies. For instance, an option could involve transmembrane regulatory proteins associated to AMPA receptors (briefly, TARPs; see Milstein and Nicoll, 2008). In particular, it has been shown that TARP-γ2, which is expressed in the majority of neuronal types in the cerebellum (Coombs and Cull-Candy, 2009), contributes to trafficking, subunit expression and functional properties of AMPA receptors (Menuz et al., 2008; Menuz and Nicoll, 2008); even more interestingly, TARP-γ2 has been shown to contribute to the amplitude of CF-EPCS in PCs (Yamazaki et al., 2010).

The observation that currents mediated by AMPA and NMDA receptors are affected in the same way in hippocampus and cerebellum suggests that enhanced excitation and compromised synaptic plasticity are generalized disturbances affecting brain structures lacking Dp71. We previously showed that *Dp71*-null mice display deficits in spatial learning and spatial position recognition memory, which have been attributed to hippocampal dysfunction (Daoud et al., 2009b). However, the present alterations in cerebellar neurophysiology prompted us to further characterize cerebellar functions at the behavioral level. The cerebellum has a well-known role in the control of balance and posture and also in learning of motor tasks. We therefore started by evaluating cerebellum integrity in a detailed characterization of motor functions, including muscle strength, ambulation in novel environment, basal static and dynamic coordination in rotarod, performance in hole board, unstable platform and traction reflex tests, and fine-timing motor learning in the rotarod. In line with the lack of function for Dp71 in skeletal muscles, *Dp71*-null mice showed no impairment in tests evaluating sustained neuromuscular strength. More surprisingly, *Dp71*-null mice did not display any motor impairment. There were no spontaneous dystonic postures, crawling or motor stereotypies and the mice were not obviously ataxic during normal walking. This observation excludes the presence of a main cerebellar dysgenesis, such as reported in spinocebellar mutant lines, or major dysregulation of muscle tone involving dysfunctional cerebellar anterior vermis (Aruga et al., 2004). No deficits were found when mice were tested for equilibrium on a static rod or in different paradigms requiring vestibular-dependent processing to maintain equilibrium or more strict motor coordination during motion, such as during the wire-suspension and rotarod synchronization learning tasks. Moreover, *Dp71*-null mice did not express any disinhibitory tendencies, such as those observed in mouse models of cerebellar dysfunctions (Rondi-Reig et al., 1999; Lalonde and Strazielle, 2007); therefore, we confirm that mice lacking Dp71 do not phenocopy mouse models of cerebellar degeneration, which commonly display major alterations in motor coordination tasks and disinhibitory tendencies. Thus, despite the obvious presence of neurophysiological alterations in CF inputs onto PCs, cerebellum-dependent motor functions appear to be largely preserved in mice lacking Dp71, suggesting the putative involvement of compensatory mechanisms. Such a possibility has been illustrated by studies showing, for instance, that changes at CF-PC synapses can be compensated for at the level of PF-PC synapses by several types of plasticity that do not require CF activity for induction and are important for motor learning (Ohtsuki et al., 2009).

Nevertheless, we have also identified selective behavioral alterations in *Dp71*-null mice, which suggest a contribution of Dp71 to cerebellar functions. First, *Dp71*-null mice outperformed WT mice during coordination learning in the traction reflex and rotarod tests. As a reduction of CF-EPSC is accompanied by impaired motor performance in rotarod and traction reflex in mouse models that selectively target PCs, one might suggest that the enhanced transmission at CF-PC synapses could explain these discrete but significant facilitations of motor learning in *Dp71*-null mice (Yamazaki et al., 2015). By contrast, we also show here that *Dp71*-null mice display learning delays and alterations of behavioral strategies during non-spatial navigation tasks, suggesting that the synaptic alterations in this model could alter selective cerebellum-dependent cognitive functions. Indeed, the past decades have largely challenged the traditional view of the cerebellum as being exclusively devoted to coordinate motor function. The role of cerebellar networks has been extended to the modulation of cognitive, executive and affective processing, including a role in non-motor conditions such as autism spectrum disorders (Reeber et al., 2013; for a review). A main cognitive ability reported to be affected by cerebellar deficits is the capacity to acquire an efficient strategy in a given context to solve a spatial problem. Cerebellar circuitry acts as an adaptive filter of sensory information, linking the spatial context and the motor response characterized by the animal's trajectory to optimize motor response during navigation and in self-motion-based hippocampal representation and path integration (Burguière et al., 2005, 2010). Accordingly, rodent models of cerebellar degeneration are greatly impaired in processing the procedural components of spatial events, such as in goal-oriented tasks involving navigation strategies in a water maze (Steinmayr et al., 1998; Leggio et al., 1999; Rondi-Reig and Burguière, 2005; Rochefort et al., 2011). To investigate this, we used two goal-oriented tasks that minimize hippocampus-dependent spatial processing: (1) a navigation task with a non-visible platform and a fixed start–fixed arrival procedure, in which mice learn to orient their body directly to the platform, and (2) a pure visuomotor guidance task in which mice can use a single cue (visible platform) to reach the platform (Table 1). Both partial and total lesion of CF and PF inputs of the cerebellum have been shown to induce deficits in the non-visible platform task, but not with a visible platform (Rondi-Reig et al., 2002). We therefore hypothesized that the alterations of CF synapses in *Dp71*-null mice would selectively alter their performance in the non-visible platform task. Indeed, *Dp71*-null mice displayed a learning delay during the period normally needed to quickly acquire the procedural component of the task. Moreover, they displayed a less precise motion direction when engaged in a search strategy to find the hidden platform, which further suggests difficulties to express optimal trajectories during path integration. This difference could not be attributed to changes in their motivation to escape and/or ability to swim in high motor-demand aversive situations, as performance of the two genotypes were comparable in behavioral-despair tests. Despite their initial navigation deficit in the non-visible platform task, *Dp71*-null mice had no impairment during the next days of testing and finally learnt the platform position, thus showing a much less severe phenotype than mouse models with cerebellar degeneration or impaired cerebellar connectivity. As expected, however, *Dp71*-null mice had deficits selectively in the fixed start–fixed arrival procedure condition, not in the visible-platform condition, which further supports evidence that changes in PC physiology specifically altered cerebellum-dependent navigation, but not simple visuomotor guidance.

In conclusion, our results demonstrate that the absence of Dp71 in mice is associated with changes in the expression of scaffolding postsynaptic proteins, enhanced excitatory neurotransmission and impaired synaptic plasticity at CF synapses to Purkinje neurons in the spinocerebellum. This is unequivocally consistent with what we observed in other structures, such as hippocampus (Daoud et al., 2009b) and cortex (C.V., personal observations), and suggest that enhanced excitation and impaired plasticity probably constitute a main, and general, feature of brain structures that lack Dp71. However, we also provide evidence that cerebellar functions are mostly preserved at the behavioral level, the main outcomes of Dp71 loss being selective changes in navigation strategies that might contribute to spatial-learning impairments and discrete improvements of performance in motor learning. These results are particularly relevant regarding the debated emphasis placed on cerebellar dysfunction in clinical studies of DMD patients, which either pointed to cerebellar dysfunctions based on the comparison of patient's cognitive profile with theoretical anatomo-functional models (Cyrulnik and Hinton, 2008) or, by contrast, did not detect any deficit in specific cerebellum-dependent tasks (Schara et al., 2015). Moreover, these studies were both performed in a heterogeneous population of patients holding distinct mutations without providing genotype-phenotype data to determine which dystrophin isoform was involved. Hence, the importance of deciphering cerebellar functions in distinct mouse models holding specific mutations in the dystrophin gene. A role for the full-length Dp427 dystrophin in cerebellar functions has been suggested by studies of *mdx* mice (Grady et al., 2006; Anderson et al., 2010), yet muscle wasting in this model lessens interpretations at the behavioral level. Our study provides the first evaluation of the role of Dp71 in cerebellar functions using a mouse model that displays a selective loss of this protein but no myopathy. Our results demonstrate the presence of cellular alterations in a crucial pool of PC synapses, which result in selective and moderate behavioral modifications in mice. The learning deficits of *Dp71*-null mice in navigation tasks are more important in conditions that require hippocampal-dependent processing of spatial information (Daoud et al., 2009b), suggesting a variability of the functional deficits induced by Dp71 deficiency depending on the brain structure. Although the hippocampus is not required for the navigation tasks used here, we could not totally exclude its putative contribution. We therefore suggest that the cognitive and behavioral dysfunctions resulting from Dp71 loss do not rely on a single brain structure and will be better understood in terms of their interactions within larger scale brain circuits.

## MATERIALS AND METHODS

### Animals

*Dp71*-null mice were a kind gift from Professor David Yaffe (Weizmann Institute of Science, Rehovot, Israel). They were originally generated in his laboratory by homologous recombination, by replacing most of the first and unique exon and a small part of the first intron of Dp71 with the promoterless gene encoding a β-Gal-neomycin resistance chimeric protein. This manipulation specifically abolished the expression of Dp71 without interfering with the expression of other *dmd* gene products (Sarig et al., 1999). Transgenic mice were backcrossed for >10 generations to C57BL/6JRj mice (Janvier Labs, France) in CDTA (Orléans, France) and breeders were kindly provided by Dr Alvaro Rendon (Institut de la Vision, Paris, France). Production and maintenance of the transgenic line was undertaken in our animal facility by crossing heterozygous females with C57BL/6JRj mice to generate *Dp71*-null and littermate controls (WT) males for experiments. Genotype was determined by PCR analysis of tail DNA. Animals were kept under a 12 h light-dark cycle (light on 7.00 am) with food and water *ad libitum*. Studies were conducted blind to the genotype following guidelines of the local mouse facility (agreement #D91-471-104) in compliance with European Directive 2010/63/EU and French National Committee (87/848). Experimental protocols were approved by the animal welfare body of our institution (Institut des Neurosciences, Neuro-PSI) and Ethics Committee #59.

### Immunofluorescence and confocal image analyses

Sagittal cerebellar sections (30 µm thick) cut at −12°C in a cryostat from fresh-frozen dissected brains were collected on SuperFrost^TM^ glass slides (Roth, France) and stored at −80°C. For immunochemistry, slides were thawed for 1 min at room temperature, immersed in acetone/methanol (1:1) for 5 min at −20°C, washed 3 times in 0.1 M phosphate-buffered saline (PBS) and incubated in a blocking solution for 45 min [10% normal goat serum (NGS), 0.3% Triton X-100, 1% bovine serum albumin (BSA)]. Slides were then incubated overnight at 4°C with a primary polyclonal antibody directed against the postsynaptic density protein PSD-95 (1:400; Invitrogen, #51-6900) followed by washes and incubation with a goat anti-rabbit secondary antibody conjugated to Cy3 (1:500; Jackson ImmunoResearch, USA, #111-165-003) in PBS (0.1 M) with 5% NGS and 1% BSA for 1 h at room temperature. Cover slips were applied to slides using a mounting medium containing 4′,6-diamino-2-phenylindole (DAPI; Fluoromount-G, Clinisciences, France). No staining was observed in sections processed from control sections from both genotypes when primary antibody was omitted. A laser scanning confocal microscope LSM 700 (Zeiss, France) was used to sequentially collect Cy3 immunoreactivity at 555 nm and DAPI staining at 405 nm. Confocal images were imported using an EC Plan-Neofluar 40×/1.30 Oil M27 at a resolution of 156 nm/pixel. All images were randomly taken at the same exposure times and equivalent stereotaxic coordinates. Punctate immunoreactivity representing presumptive protein clusters was quantified using a threshold segmentation algorithm in WCIF ImageJ for automatic detection of clusters (Vaillend et al., 2010). Minimal cluster size was arbitrarily set to 3 adjacent pixels (0.05 µm^2^) and a maximal size of 2 μm^2^, corresponding to more than 99% of clusters. The number and size of clusters were analyzed within a total tissue surface of 13,165 µm^2^ per genotype (3 mice, >800 clusters per genotype).

### Electrophysiology

#### Tissue-slice preparation

Electrophysiological experiments were performed on C57Bl6 mice and their *Dp71*-null littermates. Male, adult (2-4 month old) mice were anesthetized with 2-bromo-2-chloro-1,1,1-trifluoroethane (Sigma-Aldrich, France), decapitated and the vermis region of the cerebellum was isolated. Parasagittal slices (250 µm thick) were prepared in the presence of a cold buffered bicarbonate solution (BBS) containing 124 mM NaCl, 3 mM KCl, 1.15 mM KH_2_PO_4_, 1.15 mM MgSO_4_, 24 mM NaHCO_3_, 10 mM glucose, 2 mM CaCl_2_ (osmolarity 330 mosmol/l; pH 7.35). Slices were then incubated at room temperature in BBS gassed with 95% O_2_-5% CO_2_ for at least 30 min before recording.

#### Electrophysiological recordings

Patch-clamp whole-cell recordings in voltage clamp mode were performed with an Axopatch 200 amplifier (Axon Instruments, Molecular Devices, USA). Pipettes were pulled from borosilicate glass capillaries and had a resistance of 5.5-6.5 MΩ when filled with the intracellular solution. For voltage clamp experiments, cells were maintained at a holding potential of Vh=−70 mV to record the response to PF stimulation (PF-EPSC), whereas to study the CF-PC synapse (CF-EPSC) the membrane potential was Vh=−20 mV. Series resistance was routinely checked delivering a negative (−10 mV) voltage step; this parameter was usually <15 MΩ, it was partially compensated (60-75%) and the recordings were interrupted whenever the series resistance increased by more than 20-30% of its initial value. During experiments, the recording chamber was continuously perfused at a rate of 2 ml/min with BBS supplemented with the GABA_A_ receptor antagonist gabazine (5 µM, Sigma-Aldrich) or bicuculline methiodide (10 µM, Sigma-Aldrich). All experiments were performed at room temperature. EPSCs were evoked by 100 µs current pulses generated by a stimulus isolator (DS2, Digitimer Ltd, UK) and delivered through a BBS-filled monopolar electrode placed in the tissue slice.

#### PF stimulation

The stimulating electrode was applied in the middle of the molecular layer and two pulses were delivered separated by an interstimulus interval of 30 ms, except in experiments examining the short-term plasticity at different interstimulus intervals. To record PF-EPSCs the patch pipette was filled with an internal solution containing 140 mM Cs-gluconate, 10 mM HEPES, 1 mM EGTA, 4.6 mM MgCl_2_, 0.1 mM CaCl_2_, 4 mM Na_2_-ATP, 0.4 mM Na_3_-GTP, and 20 mM tetraethylammonium (TEA) (final pH 7.3 with CsOH and osmolarity 300 mosmol/l). PF-EPSCs were recorded at a membrane holding potential of −70 mV. Electrical stimulation was delivered at a frequency of 0.2 Hz.

#### CF stimulation

To evoke CF-EPSCs, the stimulation electrode was placed in the granular layer. PC was first recorded at −70 mV and then at −20 mV to inactivate voltage-dependent channels and to reduce CF-EPSC amplitude and consequent space clamp problems. For this type of experiment, the patch pipette was filled with an internal solution containing 140 mM Cs-gluconate, 10 mM HEPES, 20 mM BAPTA, 4.6 mM MgCl_2_, 0.1 mM CaCl_2_, 4 mM Na_2_-ATP, 0.4 mM Na_3_-GTP and 20 mM tetraethylammonium (TEA) (final pH 7.3 with CsOH and osmolarity 300 mosmol/l). To assess the presence of one or more CF innervating the patched Purkinje neuron, several locations of the stimulating electrode in the granular layer and in the lower half of the molecular layer were systematically tested for their ability to evoke potential multistep CF-EPSCs. In rare cases, two CFs were contacting Purkinje neurons and these recordings were excluded from data analysis. In experiments studying CF-EPSC kinetics and TBOA effects, low concentrations of NBQX (0.2-0.4 µM) were added to BBS solution in order to reduce voltage-clamp escape owing to the large amplitude CF-EPSCs (holding potential=−70 mV). In voltage clamp experiments, CFs were stimulated at 0.2 Hz.

#### Recording complex spikes and synaptic plasticity induction

For current clamp experiments and plasticity induction at CF-PC synapse, patch pipettes were filled with an internal solution containing: 137 mM K gluconate, 6 mM KCl, 3.5 mM MgCl_2_, 10 mM HEPES, 4 mM NaCl, 4 mM Na_2_-ATP, 0.4 mM Na_3_-GTP (pH adjusted to 7.3 with KOH; osmolarity 295 mosmol/l). The resting membrane potential of PCs was held to −65/­−59 mV by injection of small negative currents through the amplifier (Axopatch 200B, Axon Instruments) and series resistance were routinely controlled switching to voltage clamp mode. CFs were stimulated at a frequency of 0.033 Hz, CF-mediated excitatory postsynaptic potentials with superposed complex spikes were recorded for at least 5 min in the presence of gabazine before long-term plasticity induction. For this purpose, CFs were stimulated at 5 Hz for 30 s, as previously described (Hansel and Linden, 2000). To monitor complex spike modifications, the amplitude of spike components was measured for each experiment and then normalized to the mean amplitude before plasticity induction. Normalized spike amplitudes were then averaged over several cells. Statistics were performed on averaged amplitude values over a 5 min period.

#### Paired-pulse ratio

The PPR was calculated for groups of 30 consecutive sweeps as the ratio of the mean amplitude of the second EPSC to the mean of the first EPSC. Data from different experiments were averaged to obtain the PPR mean values.

#### Data recording and analysis

Data were collected by Elphy software (G. Sadoc, CNRS, UNIC, France). CF- and PF-mediated responses were analyzed off-line by using Clampfit (Axon Instruments) and Igor (Wavemetrics, USA) routines.

### Behavioral phenotyping test battery

#### Exploration and motor coordination in a hole board

The apparatus was derived from one previously used with cerebellar mutant mice (Guastavino, 1984; Steinmayr et al., 1998; Rondi-Reig et al., 1999) and was customized by Ugo Basile (Italy) following our recommendations. The apparatus consists of an experimental box (35×35×25 cm) with a raised (2 cm) platform, in which 36 holes were drilled (2 cm in diameter, spaced by about 4 cm) and arranged in a 6×6 array. The mouse was placed in the center of the platform and video-tracked for 5 min using the ANY-maze software (Stoelting, USA) for automatic quantification of distance travelled and travelling speed. The number of times the mouse stumbled (one leg slipping into a hole) or performed hole nose pokes was manually recorded using event-recorder keys. Stumble number was considered as a measure of motor coordination and nose pokes as an estimation of exploratory stereotypies. A central square containing a 4×4 hole array was used to assess avoidance of the central anxiogenic area.

#### Grip strength test

Strength was evaluated using a home-made traction apparatus equipped with a horizontal grid to allow for gripping, connected to a digital dynamometer. Each mouse was lifted by the tail, lowered over the top of the grid and allowed to grasp the grid with both forepaws and hindpaws. While the torso of the animal was kept parallel to the grid, the mouse was gently pulled back by the tail until it released its grip and the maximum tension (arbitrary unit) was recorded. Three measures taken with a 5 min intertrial interval (ITI) were averaged.

#### Inverted screen test

Mice were placed individually on a cage wire screen about 35 cm above a table. After slowly inverting the screen upside down to 180° the ability to maintain a grip was monitored (grip latency) and a maximum score of 120 s given if the animal did not fall. Testing was repeated three times with a 5 min ITI.

#### Wire suspension test

The mouse was hung by the forepaws on a 25 cm wire (3 mm in diameter) resting on two vertical supports and elevated 35 cm above a flat surface. Three trials spaced by a 5 min pause were performed with each trial limited to a 60 s duration. The amount of time spent by the mouse hanging onto the wire and the latency to touch the wire with one hind paw were recorded during each trial; a mean score was then calculated. Other qualitative parameters were recorded and a score was attributed corresponding to the best performance achieved within the minute of testing according to the following scale (Aruga et al., 2004): 0, fell off; 1, clung to the bar with two forepaws; 2, attempted to climb on to the bar besides clinging to it with two forepaws; 3, hung on to the bar with two forepaws and one or both hind paws; 4, hung on to the bar with all four paws with the tail additionally wrapped around the bar; 5, escaped to one of the supports.

#### Static balance on an unstable platform

The apparatus consisted of a circular platform (diameter 8.5 cm) made of grey Perspex; the platform was fixed at its center on a vertical axis (1 m high) and could tilt by 30° in every direction. The mouse was placed on the middle of the platform (horizontal situation) and the number of slips per min (at least one paw out of the platform circumference) and the fall latency (cut-off 180 s) were measured by direct observation during three trials with an ITI of 15 min. The day after, the ability of mice to maintain a balance using vestibular-associated self-motion cues was assessed by submitting the mice to one trial in total darkness (visual cue removal) (Rochefort et al., 2011).

#### Forced swim test

Each mouse was lowered in an inescapable glass cylinder (diameter 11 cm, height 23 cm) filled with water to a depth of 18 cm at 25°C. Room temperature was 25°C. Behavior was recorded on video for 5 min each day in two sessions separated by a 24 h delay. Video footage was analyzed offline using event-recorder keys in Any-maze to quantify the latency and duration of three main parameters: climbing, staying afloat and immobility (freezing). Climbing was considered when mice had a vertical position of the spine with the forepaws striking the glass walls, while hind paws showed repetitive movement in the water. Staying afloat corresponded to movements simply performed to keep the head above water. Immobility was defined by a complete immobilization of the body for at least 1 s. The time not spent performing any one of these activities represented either unspecific uncoordinated movements or swimming activity involving horizontal spine position with legs treading water and producing a clear displacement of body.

#### Tail suspension test

Each mouse was suspended by adhesive tape placed 2 cm from the tip of the tail, 35 cm above a bench top during a 6 min period. Behavior was recorded on video during two sessions separated by a 24 h delay. The latency to the first bout of immobility (freezing latency) and the duration of freezing were quantified offline using event-recorder keys in Any-maze. Complete immobility for >2 s was regarded as freezing.

#### Rotarod test paradigms

Motor coordination and learning were evaluated by using several paradigms in a mouse Rotarod with adjustable speed and accelerating mode (Cat #47600, Ugo Basile, Italy). Four to five mice from each of the two genotypes were tested in parallel between 9 am and 1 pm; the apparatus was cleaned with 100% ethanol after each trial. On the first day, equilibrium was tested by placing each mouse on the non-rotating rod, its body axis perpendicular to the rod longitudinal axis. The time the animal stayed on the rod was recorded and the trial stopped when the animal fell or after 180 s. On the next day, motor coordination learning was evaluated by placing each mouse on the rod rotating at a constant speed (4 rpm), its head directed against the direction of rotation so that it had to progress forward and synchronize its walk with the speed of the rod to maintain balance. Training consisted of five successive trials with a 15 min ITI; fall latency was recorded during each trial with a 180 s cut-off duration. Motor synchronization learning was further analyzed 72 h later by placing mice on the rotating rod, which accelerated from 4 to 40 rpm in a 5 min trial. Training then consisted of five successive trials per day with 1 min ITI, during 4 successive days. A distinct group of naïve mice (both genotypes) was tested in a different protocol (Galante et al., 2009), to assess motor synchronization learning without prior experience of either motor tests or familiarization to rotarod. In this experiment, mice were submitted to 5 min trials during which the rotating rod accelerated from 4 to 40 rpm. Mice underwent four trials per day with a 1 h ITI during 3 days. On the next day (day 4), the mice were submitted to eight consecutive 2 min trials at constant speeds of 40, 35, 30, 25, 20, 15, 10 and 5 rpm, respectively. This protocol was repeated four times with a 1 h ITI.

#### Navigation tasks in a water maze

The maze was a circular water tank (1.5 m diameter) placed in a well-lit room containing several extramaze cues on the walls and surmounted by a video camera connected to a computer located in an adjacent room. The maze was filled with water (22°C) to 15 cm below the edge of wall, which was made opaque by the addition of a white paint (Opacifier 631, Morton SA, France), and it contained a circular escape platform (10 cm diameter) submerged 0.5 cm below the water surface. Four positions (N, S, E and W) equidistantly located around the maze divided it into four virtual quadrants. During pretraining (day 1), the platform was laid at the north (35 cm from the wall). The mouse was released in the center of the maze and could swim for 60 s, after which it was gently guided by hand to the platform and allowed to remain on it for 60 s. Two distinct groups of mice (both genotypes) were then tested following two distinct protocols: the visible (cued) and non-visible platform tasks. In the first protocol, a visible black tube (10 cm high) was placed on top of the platform as a beacon enabling visual guidance strategies (proximal cue). In each trial the platform was placed in the center of a different quadrant (35 cm from the wall) and the mouse was introduced in the maze from different starting points (avoiding the quadrant where the platform was located). Each mouse was submitted to four trials a day with a 10 min ITI over 3 days, during which it was allowed to swim freely for 90 s to find the platform and could stay on it for 60 s. In the non-visible platform task, single constant start point and platform position were assigned to each mouse. Each mouse underwent four 90 s trials a day with a 10 min ITI during 7 days. In both tasks, mice were videotracked using the ANY-maze software (Stoelting, USA) for automatic quantification of averaged and maximum swim speed, time needed to reach the platform (escape latency) and distance swum to the platform. Other parameters were analyzed to detail the animal's navigation strategy: entries in the quadrant containing the platform, amount of circling behavior in a virtual corridor 19 cm in width set along the wall (thigmotaxis), 360° rotations of the animal's body, absolute turn angle between each movement vector of the mouse, cumulative product of the distance from platform and time spent at this distance (cumulative distance to platform), meander (cumulative distance divided by total distance), and angular velocity (cumulative distance divided by escape latency). We also analyzed indexes reflecting deviation between optimal direction towards the platform and actual motion direction, such as angular deviation (heading error) from platform position, corrected integrated path length, path efficiency, and percentage time spent and distance swum in a 15 cm wide virtual corridor that runs from the animal's start position to the platform.

### Statistics

Data are expressed as mean±s.e.m. Behavioral parameters were analyzed using two-way ANOVA tests with one between-subject factor (genotype: WT or *Dp71*-null) and one dependent variable or within-subject factor when appropriate (training day, trial, rotating speed). For electrophysiology data, genotype effects were evaluated with the Mann-Whitney test and drug effects within groups with the Wilcoxon signed rank test. For western blot analyses, statistical significance was evaluated by the Mann-Whitney non-parametric test. *P*-values <0.05 were considered statistically significant. The Kolmogorov-Smirnov test was used to compare the distribution of protein cluster sizes (significance threshold *P<*0.005).
